# PRIMPOL promotes replication fork progression but not double strand break formation in FBH1-deficient cells in response to hydroxyurea

**DOI:** 10.1101/2025.07.08.663736

**Published:** 2025-07-12

**Authors:** Joshua L. Turner, Georgia Moore, Jennifer M. Mason

## Abstract

In response to stress, stalled replication forks undergo fork reversal converting replication forks into a 4-way junction. FBH1 is a DNA helicase that promotes replication fork reversal. After prolonged replication stress, FBH1 promotes double strand break accumulation and cell death. One of the hallmarks of loss of fork reversal is unrestrained replication in the presence of DNA damage, but how FBH1 restrains replication at stalled forks is not known. Here, we show that FBH1 limits replication fork progression by PRIMPOL in response to hydroxyurea. However, PRIMPOL is not preventing double strand break formation in FBH1 knockout cells. Finally, we demonstrate that increased resistance of FBH1-deficient cells to hydroxyurea treatment is not due to unrestrained replication by PRIMPOL. Our results indicate FBH1 restrains PRIMPOL-mediated DNA synthesis at stalled replication forks and promoting double strand break accumulation occur by distinct mechanisms.

## Introduction

Replication forks encounter impediments that slow or stop replication fork progression threatening genome integrity. Prolonged replication fork stalling results in double strand break formation (aka replication fork collapse). To protect the integrity of DNA during replication, cells have evolved complex mechanisms involving DNA damage signaling, DNA repair pathways, and DNA damage tolerance (DTT) pathways collectively referred to as the replication stress response to resolve underlying damage to allow for the completion of replication (1).

Fork reversal, a process that converts a 3-way junction into a 4-way junction by re-annealing of nascent DNA strands is crucial for the replication stress response (2). Two independent fork reversal pathways exist in mammalian cells, one pathway is mediated by HLTF, SMARCAL1, and ZRANB3 and a second pathway is mediated by FBH1 (3–6, 7). Both fork reversal pathways require RAD51 and RAD54, however, only the FBH1-dependent pathway requires the branch migration activity of RAD54L (3,8,9). Fork reversal protects replication forks from nucleolytic degradation and facilitates fork restart. RAD51 and fork protection proteins including BOD1L, FANCD2 and BRCA2 prevent degradation by nucleases including MRE11 (10–12). A second function of fork reversal is to limit the activity of potentially error-prone DNA damage tolerance (DTT) pathways from continuing replication at stalled forks. In the absence of fork reversal mediated by HLTF, SMARCAL1, RAD51, and RAD54, replication forks exhibit unrestrained replication in response to stress (9,13–15). Several studies have shown that unrestrained replication is due to increased activity of DDT pathways that bypass lesions to continue replication (13,14,16,17). The HLTF-mediated pathway has been shown to restrain replication by limiting PRIMPOL-mediated repriming (13). PRIMPOL is a primase-polymerase that can reprime downstream leaving a ssDNA gap behind the fork. In a cell line expressing a HIRAN mutant of HLTF, unrestrained replication was mediated by translesion synthesis (TLS) indicating fork reversal may inhibit additional DDT pathways. The role of the HLTF-mediated pathway has been extensively studied, but much less is known about how FBH1-mediated fork reversal limits DDT pathways at stalled replication forks.

In addition to fork reversal, FBH1 cooperates with the endonuclease MUS81 to promote double strand break (DSB) formation and signaling in response to prolonged stress (18,19). FBH1 loss is associated with decreased DSB-dependent signaling including pRPA S4/8, pan-nuclear $\gamma$H2AX, and pP53 S15 (18–21). FBH1-deficiency results in increased resistance to replication stress including ultra-violet (UV) radiation and hydroxyurea (HU) (18–20). The function of FBH1 in the replication stress response has an anti-tumorigenic role. FBH1 is deleted or mutated in a high frequency of melanoma cell lines and protects melanocytes from UV-mediated transformation (20).

Given increased PRIMPOL activity has been associated with increased chemoresistance, we determined how FBH1 restrains replication progression at forks stalled by HU. Here, we show FBH1 restrains PRIMPOL-mediated DNA synthesis after treatment with HU. However, we determined PRIMPOL is not contributing to reduced HU-induced DSB formation or increased HU resistance in FBH1-deficient cells. Together, these results indicate FBH1-mediated fork reversal limits PRIMPOL activity at replication forks, but increased replication stress resistance in FBH1 deficient cells is independent of PRIMPOL-mediated fork progression.

## Results

### PRIMPOL facilitates unrestrained fork progression in FBH1-deficient cells

Loss of fork reversal in cells deficient of HLTF, SMARCL1, or ZRANB3 leads to unrestrained fork progression during induced replication stress (13,14). FBH1 also promotes fork reversal and promotes slowing of the replication fork in the presence of HU (7). Previous studies have shown PRIMPOL-repriming mediates replication in the cells lacking fork reversal mediated by HLTF and SMARCAL1 (13,14). To determine whether PRIMPOL was promoting replication in FBH1 KO cells, we used the replication fiber assay to measured DNA tract lengths in the presence of HU in cells after depletion of PRIMPOL **(Fig. 1A).** We sequentially pulsed cells with the nucleotide analogs, CldU and IdU. During the secondary IdU pulse, we introduced a low dose of HU to slow replication fork progression **(Fig. 1B)**. In U2OS cells, PRIMPOL knockdown (KD) did not significantly change nascent tract length compared to U2OS cells **(Fig. 1C).** In FBH1 KO cells, PRIMPOL KD rescued unrestrained fork progression, reducing replication progression to the lengths observed in U2OS cells. We verified this result in RPE-1 cells after co-depletion of PRIMPOL and FBH1 (**Fig. 1D, E**). As observed in U2OS, FBH1-depletion alone resulted in unrestrained replication fork progression in the presence of HU and progression was rescued by depletion of PRIMPOL. Together, these results indicate that FBH1 inhibits unrestrained fork progression by PRIMPOL.

### PRIMPOL does not restore double strand break formation in FBH1-deficient cells

Prolonged replication stress leads to exhaustion of cellular pools of RPA resulting in wide-spread DSB accumulation (i.e., replication fork collapse) known as replication catastrophe (22). Replication catastrophe is marked by high levels of RPA phosphorylation at serine 4 and 8 (pRPA S4/8) and serine 139 on H2AX (γH2AX). FBH1-deficient cells exhibit reduced DSB accumulation and signaling after prolonged replication stress induced by HU (18–21,23). Previous studies have shown that PRIMPOL-mediated repriming limits ssDNA accumulation and increased PRIMPOL activity is associated with increased chemoresistance in BRCA1-deficient cells (13,14). Thus, we determined whether PRIMPOL-mediated fork progression is resulting in reduced DSB formation and signaling in FBH1 KO cells. First, we measured the percentage of pRPA S4/8 positive cells after treatment with HU **(Supplementary Fig. 1A).** PRIMPOL KD in U2OS cells displayed a slightly higher percentage of pRPA S4/8 positive cells than U2OS transfected with a non-silencing siRNA (siNS). Consistent with previous studies, FBH1 KO significantly reduced the percentage of cells positive for pRPA S4/8. PRIMPOL KD in FBH1 KO cells significantly increased pRPA S4/8 positive cells compared to FBH1 KO alone **(Supplementary Fig. 1B)**. This result suggest PRIMPOL KD is restoring DSB signaling in FBH1-deficient cells. However, we noticed pRPA S4/8 staining in PRIMPOL KD alone appeared more intense than U2OS or when combined with FBH1 KO. This led us to hypothesize that PRIMPOL was increasing pRPA S4/8 induction independently of FBH1 status. To test this, we measured staining intensity of pRPA S4/8 in RPA positive cells using high-throughput microcopy (**Fig. 2A**). In U2OS cells, PRIMPOL KD resulted in a significant increase in pRPA S4/8 intensity compared to siNS controls indicating PRIMPOL KD is increasing DSB signaling in

U2OS cells **(Fig. 2A).** In FBH1 KO cells, we observed a significant decrease in pRPA S4/8 intensity compared to U2OS cells. PRIMPOL KD in FBH1 KO cells slightly increases pRPA S4/8 intensity compared to FBH1 KO alone; however, this increase is consistent with the level observed by knocking down PRIMPOL alone in U2OS cells. We observed a similar result when we co-depleted FBH1 and PRIMPOL in RPE-1 cells (**Supplementary Fig. 2A**). Thus, we conclude that FBH1 KO reduces DSB signaling in PRIMPOL KD and the loss of DSB signaling in FBH1 KO cells is PRIMPOL independent.

Next, we looked at γH2AX intensity in PCNA-positive cells (i.e., S phase) in FBH1 KO cells after PRIMPOL depletion **(Fig. 2B).** As expected, FBH1 KO cells exhibited a significant reduction in γH2AX intensity compared to U2OS cells alone. Similar to the pRPA S4/8 results, PRIMPOL KD in FBH1 KO cells resulted in a slight increase in γH2AX, but to a similar extent as observed by PRIMPOL KD alone. We obtained a similar result in RPE-1 cells co-depleted of FBH1 and PRIMPOL **(Supplementary Fig. 2B).** As observed in U2OS, FBH1 KD resulted in a significant reduction in pRPA S4/8 and γH2AX signaling after HU treatment. PRIMPOL KD resulted increased in pRPA S4/8 and γH2AX signaling in both U2OS and FBH1 KO suggesting the increase in signaling observed in PRIMPOL KD occurs independently of FBH1 KO. These results indicate the defect in DSB signaling in FBH1-deficient cells is not due to PRIMPOL-mediated DNA synthesis.

Next, we determined how PRIMPOL depletion impacted DSB formation using the neutral COMET assay **(Fig. 2C, D)**. In U2OS cells, treatment with HU significantly increased DSB formation and PRIMPOL depletion led to a slight, but insignificant increase in DSB accumulation. As expected, FBH1 KO cells exhibited a significant reduction in DSB accumulation compared to U2OS cells (18). In FBH1 KO cells, PRIMPOL KD did not significantly increase DSB accumulation compared to FBH1 KO alone. Together, these results indicated the defect in DSB formation at HU stalled forks in FBH1-deficient cells is PRIMPOL-independent.

**Increased PRIMPOL activity does not contribute to increased cellular resistance of FBH1-deficient cells to HU**

FBH1-deficent cells exhibit increased resistance to replication stress induced by HU (18,19). PRIMPOL-mediated DNA synthesis increased resistance of BRCA1-deficient cells to cisplatin (14). To determine if PRIMPOL was leading to increased replication stress resistance in FBH1 KO cells, we measured survival of FBH1 KO cells with and without PRIMPOL KD after increasing concentrations of HU **(Fig. 3)**. Compared to U2OS, PRIMPOL depletion slightly increases sensitivity to HU. As expected, FBH1 KO cells exhibited significantly increased survival after HU treatment consistent with a role of FBH1 in promoting sensitivity to replication stress. The knockdown of PRIMPOL had no effect on cell survival in FBH1 KO cells after HU treatment. This result indicates that increased PRIMPOL-mediated replication at stalled replication forks is not resulting in increased resistance of FBH1-deficient cells to HU.

## Discussion

In this study, we demonstrate that FBH1 restrains replication at stalled replication forks. Furthermore, we show replication progression at stalled forks in the absence of FBH1 is mediated by PRIMPOL. Surprisingly, an increase PRIMPOL-mediated replication did not contribute to decreased DSB formation or signaling in FBH1-deficient cells in response to prolonged treatment with HU. Finally, PRIMPOL-depleted FBH1 KO cells exhibit increased resistance to replication stress similar to FBH1 KO cells alone. Thus, FBH1 limits PRIMPOL activity at stalled replication forks, but increased PRIMPOL-mediated DNA synthesis does not prevent cleavage of stalled replication forks in FBH1-deficient cells.

Fork reversal and PRIMPOL-mediated repriming are two alternative DTT pathways that allow cells to tolerate replication stress. In the absence of fork reversal mediated by HTLF, RAD51, and SMARCAL1, replication forks continue to progress due to increased PRIMPOL-dependent synthesis (10,13). Here, we demonstrate that FBH1 limits PRIMPOL-mediated replication at stalled replication forks indicating limiting PRIMPOL-mediated repriming is a global function of fork reversal. However, it is still unclear if the reversed fork is inhibiting PRIMPOL, or if the presence of fork reversal enzymes is sufficient to block PRIMPOL from accessing DNA to promote replication. Cells expressing a HLTF-HIRAN mutant that lacks fork reversal activity was able to block replication fork progression by PRIMPOL suggesting the presence of HLTF at stalled forks was sufficient to prevent PRIMPOL (13). FBH1 is a 3’-5’ helicase and is acting on the lagging DNA strand (24). In contrast, PRIMPOL-mediated repriming occurs on the leading strand (25). Therefore, we propose the ability of FBH1 to prevent PRIMPOL-mediated repriming is likely dependent on formation of the reversed fork and not a result of FBH1 blocking PRIMPOL access to DNA.

Our data indicate that the role of FBH1 in promoting sensitivity to HU-induced replication stress is PRIMPOL-independent. First, we show that PRIMPOL depletion does not restore DSB accumulation or DSB-dependent signaling in FBH1-deficient cells after treatment with HU. Second, PRIMPOL depletion did not rescue increased resistance of FBH1-deficient cells to HU. Thus, the role of FBH1 restraining replication by PRIMPOL and promoting sensitivity to HU are mechanistically distinct.

There are multiple potential mechanisms by which FBH1 can promote replication fork collapse after prolonged replication stress. First, FBH1-mediated fork reversal may be required to generate an intermediate that is susceptible to cleavage by nucleases. Previous work has demonstrated that reversed forks can be processed and cleaved by structure-specific endonucleases (26,27). However, we do not favor this model because loss of the fork reversal proteins, SMARCAL1 and ZRANB3, results in increased sensitivity to a variety of DNA damaging agents including mitomycin C, HU and cisplatin suggesting inhibiting fork reversal alone is not sufficient to prevent replication fork collapse (5,28–30). Second, FBH1 may limit replication fork progression by additional DDT pathways such as translesion synthesis. Previous work has demonstrated FBH1 loss results in increase Polη focus formation at UV-induced lesions (31). In this case, replication progression by additional DDT pathways may be sufficient to limit ssDNA accumulation and break formation in FBH1-deficient cells. Third, FBH1 negatively regulates homologous recombination by removing RAD51 from DNA and promoting ubiquitination of RAD51 to prevent rebinding to DNA (21,24,32). Expression of a ubiquitin-resistant RAD51 mutant phenocopied FBH1-deficient cells and exhibited increased resistance to HU (21). However, it is not known how these mutations impact RAD51 biochemical activity, or if other functions of FBH1 contribute to promoting replication stress sensitivity. Alternatively, FBH1 may prevent replication fork collapse by a mechanism independent of replication fork reversal. FBH1 cooperates with MUS81 to induce DSBs at stalled replication forks (19). FBH1 could be required for efficient localization of MUS81 to the stalled replication fork. Future work will be conducted to determine how FBH1 promotes DSB formation at stalled replication forks.

FBH1 loss has been linked to melanoma and mutation or loss of FBH1 may be present in additional tumors (20,33). An increase in PRIMPOL-mediated synthesis would give FBH1-deficient cells a proliferative advantage by allowing cells to continue replication in the presence of DNA damage. Cancer cells have high levels of replication stress due to increased proliferation and oncogene expression (34,35). However, PRIMPOL is error-prone, so the increased PRIMPOL activity at stalled forks is likely mutagenic (36).

Targeting DNA gaps left by PRIMPOL-mediated DNA synthesis may be a viable chemotherapeutic strategy to kill FBH1-deficient tumors. Single-stranded gap accumulation by PRIMPOL requires gap filling by template switching or TLS in late S-phase and G2 before mitotic entry (17). The accumulation of gaps in BRCA-deficient cells contributes to cisplatin sensitivity (37). Furthermore, TLS reduces replication stress in cancer cells by suppressing DNA gap accumulation. Cell lines dependent on gap filling are sensitive to TLS alone or in combination with ATR and WEE1 inhibition that induce DNA gaps (38). Consistent with this, work from our lab showed that FBH1 KO cells were sensitive to WEE1 inhibition due increased mitotic catastrophe (23). We propose that the sensitivity of FBH1 KO cells to WEE1 inhibitors is a result of cells being forced into mitosis with DNA gaps.

In conclusion, we demonstrate FBH1 restrains replication by PRIMPOL in response to HU-induced replication stress. However, we demonstrate PRIMPOL is not contributing to the defect in DSB accumulation or increased HU resistance observed in FBH1-deficient cells. Thus, FBH1 restrains PRIMPOL and promotes replication fork collapse by two independent mechanisms. Additional work being conducted will determine how increased PRIMPOL-dependent replication can be exploited as a chemotherapeutic strategy to kill FBH1-deficient tumors.

## Materials & Methods

### Cell culture

U2OS cells were grown in DMEM (Gibco, 1196518) supplemented with 10% FBS (Atlas Biologicals, S10350H). hTERT immortalized RPE1 cells were grown in DMEM/F12 (Corning, 10-092-CVR) supplemented with 10% FBS. Cells were grown at 37$^{o}$C in 5% $CO_2$. Hydroxyurea and cisplatin were resuspended in water and diluted in media at the indicated concentrations.

### siRNA transfections

Cells (1x10$^{5}$) were seeded in 6-well plates and allowed to adhere overnight. Transfections were prepared in 1x Opti-MEM (GIBCO) and Lipofectamine RNAiMAX (Fisher Scientific, 13778150) using manufacturer instructions. Two siRNAs against FBH1 (Thermo) were pooled and transfected at a final concentration of 75µM. The siRNA targeting PRIMPOL was from Dharmacon (J-015804). The All-Star Negative control siRNA (Qiagen) was used a non-silencing (siNS) control. siRNA sequences are:

siFBH1-1 5’ GGAGTCAGGAGACCCGGGTTCTTGT

siFBH1-2 5' CCGGGTTCTTGTGTTGGCACCATCAC

siPRIMPOL 5' GAGGAAAGCUGGACAUCGA

**Immunofluorescence**

Cells ($4x10^4$) were seeded in 12-well plates with 22mm circular cover slips and allowed to adhere overnight. Cells were treated with 2mM HU for 24 hours. Cells were permeabilized a HEPES/TritonX-100 buffer (20mM HEPES, ph7.4, 3mM $MgCl_2$, 50mM NaCl, 0.5% Triton X-100) for 10 minutes and fixed to the coverslips with ice-cold methanol. Coverslips were incubated in primary antibody overnight at $4^oC$. Primary antibodies used were PCNA (ABCAM, ab18197, 1:1000), γH2AX (ABCAM, ab26350, 1:1000), RPA (ABCAM, ab2175, 1:1000), pRPA S4/8 (Bethyl Laboratories, A300-245A-M, 1:1000). Cells were washed 3X in 1X PBS before staining with secondary antibodies. Coverslips were incubated in Alexa fluor-conjugated secondary antibodies (Invitrogen, 1:1000) for 1 hour at room temperature. Coverslips were washed 3 times for 5 minutes in 1X PBS followed by 2 minutes each in 70%, 95% and 100% ethanol and allowed to air dry. Cells were mounted on slides with vectashield containing DAPI. Cells were imaged on ImageExpress Micro 4 (Molecular Devises) and analyzed using MetaXpress software version 6.7.2.290.

**Replication fiber assay**

Cells ($4x10^4$) were seeded in 12-well plates and allowed to adhere overnight. Cells were pulsed with 20uM CldU for 30 minutes. Cells were quickly washed with 1X PBS and incubated in 75uM IdU with or without 50uM HU for 30 minutes. Cells were suspended in trypsin and resuspended in ice cold 1X PBS at $4X10^4$ cells/ml. Replication fibers were prepared as previously described (39). Slides were stained with mouse BrdU (BD, 347580, 1:50) to label IdU and rat BrdU (ABCAM, ab6326, 1:200) to label CldU. Images were acquired at 60x magnification using a Zeiss Imager.M2 epifluorescence microscope with an Axiocam 503 mono camera. The length of at least 150 replication tracts was measured using ImageJ.

**Neutral comet assay**

Cells ($2x10^5$) were seeded in 6 well plates and allowed to adhere overnight. Media was refreshed with or without 2 mM HU for 24 hours. Cells were collected and suspended in 500 µl PBS. Neutral comet assay was conducted as described with lysis buffer using comet slides and lysis buffer that were purchased from R&D Systems (4250-050-K)(40). Comets were acquired at 20x magnification using a Zeiss Imager.M2 epifluorescence microscope with an Axiocam 503 mono camera. Tail area was calculated using the open comet plugin in ImageJ (41).

**Survival assay**

Cells ($1x10^3$) were plated in triplicate in black walled 96 well plates (Corning, 3606) and allowed to adhere overnight. Cells were treated with HU for 48 hours and allowed to outgrow in fresh media for 7 days. After outgrowing live cells were stained with Hoechst 33342 (1:2000) for 20 minutes and counted using the ImageExpress Micro 4.

**Generation of FBH1 polyclonal antibodies**

The N-terminal 250 amino acids of human FBH1 were cloned in to a pGEX6p-1 expression vector using Gibson assembly. Primers used to amplify the FBH1 fragment were FBH1_pGEX6_F (5' TTCTGTTCCAGGGGCCCCTGGGATCCagctacgaggtgacttcag) and FBH1_N250_pGEX__R (5' GTCGACCCGGGAATTCCGGGGATCCtcaaagcccatagtatgagtcag). GST-FBH1[1-250] in the pGEX6P-1 vector (Amersham/GE Healthcare/Cytiva) was introduced into BL21:DE3 Rosetta cells (Novagen). The cells were grown at 37 °C to an $OD_{600}$ of 0.8 and induced with 0.4 mM IPTG for 16 h at 16 °C. All the steps of the purification were performed at 4 °C. A 10 g pellet of cells was suspended in 50 mL of cell breakage buffer (50 mM Tris-HCl, pH 7.5, 10% sucrose, 2 mM EDTA, 250 mM KCl, 0.01% IgePal, 1 mM DTT, 1 mM Phenylmethulsulfonyl fluoride, 0.5 mM benzamidine and 5 µg/mL each of aprotinin, chymostatin, leupeptin, and pepstatin). The cells were sonicated, and the crude lysate was centrifuged at 100,000 x g for 90 min. The clarified lysate was incubated with 1 mL of glutathione Sepharose (Cytiva) with constant agitation. The matrix was poured into a 1 cm column and washed with 10 mLs Buffer A (50 mM Tris-HCl, pH8.0, 10% glycerol, 1 mM EDTA, 1 mM DTT, and 0.01% IgePal) containing 1 M KCl and then 5 mL of Buffer A containing 300 mM KCl. The bound GST-FBH1[1-250] was eluted with 6 mL of 10 mM glutathione in Buffer A containing 300 mM KCl. The fractions containing GST-FBH1[1-250] were pooled and concentrated in an Amicon Ultra microconcentrator to 500 µL and subjected to size exclusion chromatography on a 40 mL Sephacryl S200 column in Buffer A containing 300 mM KCl. The fractions containing GST-FBH1[1-250] were pooled and concentrated as before to 8 mg/mL, and stored in small aliquots at -80 °C.  ~1 mg of GST-FBH1[1-250] was used to generate antibodies from Pacific Immunology.

Purified GST-FBH1[1-250] was coupled to CNBr activated Sepharose per the manufacturer's instructions.  Serum was applied to a 1 mL of the matrix in a 1 cm column.  The matrix was washed with 10 mL PBS (10 mM $Na_2HPO_4$, pH 7.4, 2.7 mM KCl, 137 mM NaCl, 0.05 % TWEEN) followed by elution with 166 mM MES pH 6.0 containing 100 mM diethylamine.  The fractions that contained the antibodies were pooled and dialyzed against PBS.  The dialyzed antibodies were concentrated in an Amicon Ultra microconcentrator, supplemented with 0.01% sodium azide and stored in small aliquots at 4 °C.

**Western Blotting**

Whole cell extracts were prepared by resuspending cells ($10^6$/100 µl) in Laemmli buffer (62.5mM Tris-HCl, ph6.8, 2% SDS, 10% glycerol, 5% 2-mercaptoethanol, and 0.002% bromophenol blue). Protein extracts were run on an SDS-Page gel and transferred to a PVDF membrane. The membrane was stained with primary antibodies recognizing FBH1(purification described above), PRIMPOL (kind Gift from Juan Mendéz (42)), and Vinculin (Cell signaling, 13901S, 1:1000) overnight at 4$^o$C. The membrane was washed 3X with TBS + 0.1% Tween-20 and then incubated with HRP secondary antibodies for 1 hour at room temperature (LI-COR, 926-80011/10, 1:2000). Membranes were incubated with WesternSure Premium Chemiluminescent substrate (Li-COR) following manufacturers' instructions. Membranes were imaged and analyzed using the LI-COR C-DiGit imager and Image Studio software version 5.2.5.

### Statistical Analysis

All experiments were conducted at least three independent times unless otherwise indicated. Statistical analyses were performed using Graphpad Prism (version 10.2.3). Significance was calculated using an ANOVA followed by Tukey HSD. Significance for the pRPA S4/8 and γH2AX intensity data was conducted on the means of each experiment. Statistical significance was determined by a *p*-value below 0.05.

## Author Contributions

JLT and JMM designed the research study. JLT and GM conducted the experiments and analyzed data. JLT and JMM wrote and edited the manuscript.

## Data availability

Further information and requests for resources and reagents should be directed to and will be fulfilled by the lead contact, Jennifer Mason, PhD (jmason4@clemson.edu). All data will be available upon request.

## Acknowledgements

We would like to thank Garrett Buzzard, Amarachukwu Onoh, and Michael Sehorn for purification of GST-tagged FBH1-N250 and FBH1 polyclonal antibodies.

## Funding

This work was funded by the American Cancer Society Research Scholar Grant (RSG-21-175-01-DMC) and NIGMS (R35 GM142512).

## References

1. Saxena, S. and Zou, L. (2022) Hallmarks of DNA replication stress. *Molecular Cell*, **82**, 2298–2314.
2. Meng, X. and Zhao, X. (2016) Replication fork regression and its regulation. *FEMS Yeast Research*, fow110.
3. Liu, W., Krishnamoorthy, A., Zhao, R. and Cortez, D. (2020) Two replication fork remodeling pathways generate nuclease substrates for distinct fork protection factors. *Sci. Adv.*, **6**, eabc3598.
4. Bétous, R., Mason, A.C., Rambo, R.P., Bansbach, C.E., Badu-Nkansah, A., Sirbu, B.M., Eichman, B.F. and Cortez, D. (2012) SMARCAL1 catalyzes fork regression and Holliday junction migration to maintain genome stability during DNA replication. *Genes Dev.*, **26**, 151–162.
5. Ciccia, A., Nimonkar, A.V., Hu, Y., Hajdu, I., Achar, Y.J., Izhar, L., Petit, S.A., Adamson, B., Yoon, J.C., Kowalczykowski, S.C., *et al.* (2012) Polyubiquitinated PCNA Recruits the ZRANB3 Translocase to Maintain Genomic Integrity after Replication Stress. *Molecular Cell*, **47**, 396–409.
6. Achar, Y.J., Balogh, D. and Haracska, L. (2011) Coordinated protein and DNA remodeling by human HLTF on stalled replication fork. *Proc. Natl. Acad. Sci. U.S.A.*, **108**, 14073–14078.
7. Fugger, K., Mistrik, M., Neelsen, K.J., Yao, Q., Zellweger, R., Kousholt, A.N., Haahr, P., Chu, W.K., Bartek, J., Lopes, M., *et al.* (2015) FBH1 Catalyzes Regression of Stalled Replication Forks. *Cell Reports*, **10**, 1749–1757.
8. Zellweger, R., Dalcher, D., Mutreja, K., Berti, M., Schmid, J.A., Herrador, R., Vindigni, A. and Lopes, M. (2015) Rad51-mediated replication fork reversal is a global response to genotoxic treatments in human cells. *Journal of Cell Biology*, **208**, 563–579.

9. Uhrig, M.E., Sharma, N., Maxwell, P., Gomez, J., Selemenakis, P., Mazin, A.V. and Wiese, C. (2024) Disparate requirements for RAD54L in replication fork reversal. *Nucleic Acids Research*, **52**, 12390–12404.
10. Schlacher, K., Wu, H. and Jasin, M. (2012) A Distinct Replication Fork Protection Pathway Connects Fanconi Anemia Tumor Suppressors to RAD51-BRCA1/2. *Cancer Cell*, **22**, 106–116.
11. Schlacher, K., Christ, N., Siaud, N., Egashira, A., Wu, H. and Jasin, M. (2011) Double-Strand Break Repair-Independent Role for BRCA2 in Blocking Stalled Replication Fork Degradation by MRE11. *Cell*, **145**, 529–542.
12. Higgs, M.R., Reynolds, J.J., Winczura, A., Blackford, A.N., Borel, V., Miller, E.S., Zlatanou, A., Nieminuszczy, J., Ryan, E.L., Davies, N.J., *et al.* (2015) BOD1L Is Required to Suppress Deleterious Resection of Stressed Replication Forks. *Molecular Cell*, **59**, 462–477.
13. Bai, G., Kermi, C., Stoy, H., Schiltz, C.J., Bacal, J., Zaino, A.M., Hadden, M.K., Eichman, B.F., Lopes, M. and Cimprich, K.A. (2020) HLTF Promotes Fork Reversal, Limiting Replication Stress Resistance and Preventing Multiple Mechanisms of Unrestrained DNA Synthesis. *Molecular Cell*, **78**, 1237-1251.e7.
14. Quinet, A., Tirman, S., Jackson, J., Šviković, S., Lemaçon, D., Carvajal-Maldonado, D., González-Acosta, D., Vessoni, A.T., Cybulla, E., Wood, M., *et al.* (2020) PRIMPOL-Mediated Adaptive Response Suppresses Replication Fork Reversal in BRCA-Deficient Cells. *Molecular Cell*, **77**, 461-474.e9.

15. Vallerga, M.B., Mansilla, S.F., Federico, M.B., Bertolin, A.P. and Gottifredi, V. (2015) Rad51 recombinase prevents Mre11 nuclease-dependent degradation and excessive PrimPol-mediated elongation of nascent DNA after UV irradiation. *Proc. Natl. Acad. Sci. U.S.A.*, **112**.
16. Kobayashi, K., Guilliam, T.A., Tsuda, M., Yamamoto, J., Bailey, L.J., Iwai, S., Takeda, S., Doherty, A.J. and Hirota, K. (2016) Repriming by PrimPol is critical for DNA replication restart downstream of lesions and chain-terminating nucleosides. *Cell Cycle*, **15**, 1997–2008.
17. Tirman, S., Quinet, A., Wood, M., Meroni, A., Cybulla, E., Jackson, J., Pegoraro, S., Simoneau, A., Zou, L. and Vindigni, A. (2021) Temporally distinct post-replicative repair mechanisms fill PRIMPOL-dependent ssDNA gaps in human cells. *Molecular Cell*, **81**, 4026-4040.e8.
18. Jeong, Y.-T., Rossi, M., Cermak, L., Saraf, A., Florens, L., Washburn, M.P., Sung, P., Schildkraut, C.L. and Pagano, M. (2013) FBH1 promotes DNA double-strand breakage and apoptosis in response to DNA replication stress. *Journal of Cell Biology*, **200**, 141–149.
19. Fugger, K., Kit Chu, W., Haahr, P., Nedergaard Kousholt, A., Beck, H., Payne, M.J., Hanada, K., Hickson, I.D. and Storgaard Sørensen, C. (2013) FBH1 co-operates with MUS81 in inducing DNA double-strand breaks and cell death following replication stress. *Nat Commun*, **4**, 1423.
20. Jeong, Y.-T., Cermak, L., Guijarro, M., Hernando, E. and Pagano, M. (2013) FBH1 protects melanocytes from transformation and is deregulated in melanomas. *Cell Cycle*, **12**, 1128–1132.

21. Fugger, K., Mistrik, M., Danielsen, J.R., Dinant, C., Falck, J., Bartek, J., Lukas, J. and Mailand, N. (2009) Human Fbh1 helicase contributes to genome maintenance via pro- and anti-recombinase activities. *Journal of Cell Biology*, **186**, 655–663.
22. Toledo, L.I., Altmeyer, M., Rask, M.-B., Lukas, C., Larsen, D.H., Povlsen, L.K., Bekker-Jensen, S., Mailand, N., Bartek, J. and Lukas, J. (2013) ATR Prohibits Replication Catastrophe by Preventing Global Exhaustion of RPA. *Cell*, **155**, 1088–1103.
23. Jennings, L., Walters, H.A., McCraw, T.J., Turner, J.L. and Mason, J.M. (2024) FBH1 deficiency sensitizes cells to WEE1 inhibition by promoting mitotic catastrophe. *DNA Repair*, **133**, 103611.
24. Masuda-Ozawa, T., Hoang, T., Seo, Y.-S., Chen, L.-F. and Spies, M. (2013) Single-molecule sorting reveals how ubiquitylation affects substrate recognition and activities of FBH1 helicase. *Nucleic Acids Research*, **41**, 3576–3587.
25. Tirman, S., Cybulla, E., Quinet, A., Meroni, A. and Vindigni, A. (2021) PRIMPOL ready, set, reprime! *Critical Reviews in Biochemistry and Molecular Biology*, **56**, 17–30.
26. Lemaçon, D., Jackson, J., Quinet, A., Brickner, J.R., Li, S., Yazinski, S., You, Z., Ira, G., Zou, L., Mosammaparast, N., *et al.* (2017) MRE11 and EXO1 nucleases degrade reversed forks and elicit MUS81-dependent fork rescue in BRCA2-deficient cells. *Nat Commun*, **8**, 860.
27. Thangavel, S., Berti, M., Levikova, M., Pinto, C., Gomathinayagam, S., Vujanovic, M., Zellweger, R., Moore, H., Lee, E.H., Hendrickson, E.A., *et al.* (2015) DNA2

drives processing and restart of reversed replication forks in human cells. *Journal of Cell Biology*, **208**, 545–562.

28. Ciccia, A., Bredemeyer, A.L., Sowa, M.E., Terret, M.-E., Jallepalli, P.V., Harper, J.W. and Elledge, S.J. (2009) The SIOD disorder protein SMARCAL1 is an RPA-interacting protein involved in replication fork restart. *Genes Dev.*, **23**, 2415–2425.
29. Yuan, J., Ghosal, G. and Chen, J. (2009) The annealing helicase HARP protects stalled replication forks. *Genes Dev.*, **23**, 2394–2399.
30. Weston, R., Peeters, H. and Ahel, D. (2012) ZRANB3 is a structure-specific ATP-dependent endonuclease involved in replication stress response. *Genes Dev.*, **26**, 1558–1572.
31. Bacquin, A., Pouvelle, C., Siaud, N., Perderiset, M., Salomé-Desnoulez, S., Tellier-Lebegue, C., Lopez, B., Charbonnier, J.-B. and Kannouche, P.L. (2013) The helicase FBH1 is tightly regulated by PCNA via CRL4(Cdt2)-mediated proteolysis in human cells. *Nucleic Acids Research*, **41**, 6501–6513.
32. Simandlova, J., Zagelbaum, J., Payne, M.J., Chu, W.K., Shevelev, I., Hanada, K., Chatterjee, S., Reid, D.A., Liu, Y., Janscak, P., *et al.* (2013) FBH1 Helicase Disrupts RAD51 Filaments in Vitro and Modulates Homologous Recombination in Mammalian Cells. *Journal of Biological Chemistry*, **288**, 34168–34180.
33. Watson, A.J., Shaffer, M.L., Bouley, R.A. and Petreaca, R.C. (2023) F-box DNA Helicase 1 (FBH1) Contributes to the Destabilization of DNA Damage Repair Machinery in Human Cancers. *Cancers*, **15**, 4439.

34. Kotsantis, P., Petermann, E. and Boulton, S.J. (2018) Mechanisms of Oncogene-Induced Replication Stress: Jigsaw Falling into Place. *Cancer Discovery*, **8**, 537–555.

35. Da Costa, A.A.B.A., Chowdhury, D., Shapiro, G.I., D'Andrea, A.D. and Konstantinopoulos, P.A. (2023) Targeting replication stress in cancer therapy. *Nat Rev Drug Discov*, **22**, 38–58.

36. Guilliam, T.A., Jozwiakowski, S.K., Ehlinger, A., Barnes, R.P., Rudd, S.G., Bailey, L.J., Skehel, J.M., Eckert, K.A., Chazin, W.J. and Doherty, A.J. (2015) Human PrimPol is a highly error-prone polymerase regulated by single-stranded DNA binding proteins. *Nucleic Acids Research*, **43**, 1056–1068.

37. Panzarino, N.J., Krais, J.J., Cong, K., Peng, M., Mosqueda, M., Nayak, S.U., Bond, S.M., Calvo, J.A., Doshi, M.B., Bere, M., *et al.* (2021) Replication Gaps Underlie BRCA Deficiency and Therapy Response. *Cancer Research*, **81**, 1388–1397.

38. Nayak, S., Calvo, J.A., Cong, K., Peng, M., Berthiaume, E., Jackson, J., Zaino, A.M., Vindigni, A., Hadden, M.K. and Cantor, S.B. (2020) Inhibition of the translesion synthesis polymerase REV1 exploits replication gaps as a cancer vulnerability. *Sci. Adv.*, **6**, eaaz7808.

39. DNA Fiber Analysis: Mind the Gap! (2017) *Methods in Enzymology*, Elsevier, pp. 55–82.

40. Roy, I., Nadar, P. and Khurana, S. (2021) Neutral Comet Assay to Detect and Quantitate DNA Double Strand Breaks in Hematopoietic Stem Cells. *BIO-PROTOCOL*, **11**.

41. Gyori, B.M., Venkatachalam, G., Thiagarajan, P.S., Hsu, D. and Clement, M.-V. (2014) OpenComet: An automated tool for comet assay image analysis. *Redox Biology*, **2**, 457–465.

42. Mourón, S., Rodriguez-Acebes, S., Martínez-Jiménez, M.I., García-Gómez, S., Chocrón, S., Blanco, L. and Méndez, J. (2013) Repriming of DNA synthesis at stalled replication forks by human PrimPol. *Nat Struct Mol Biol*, **20**, 1383–1389.

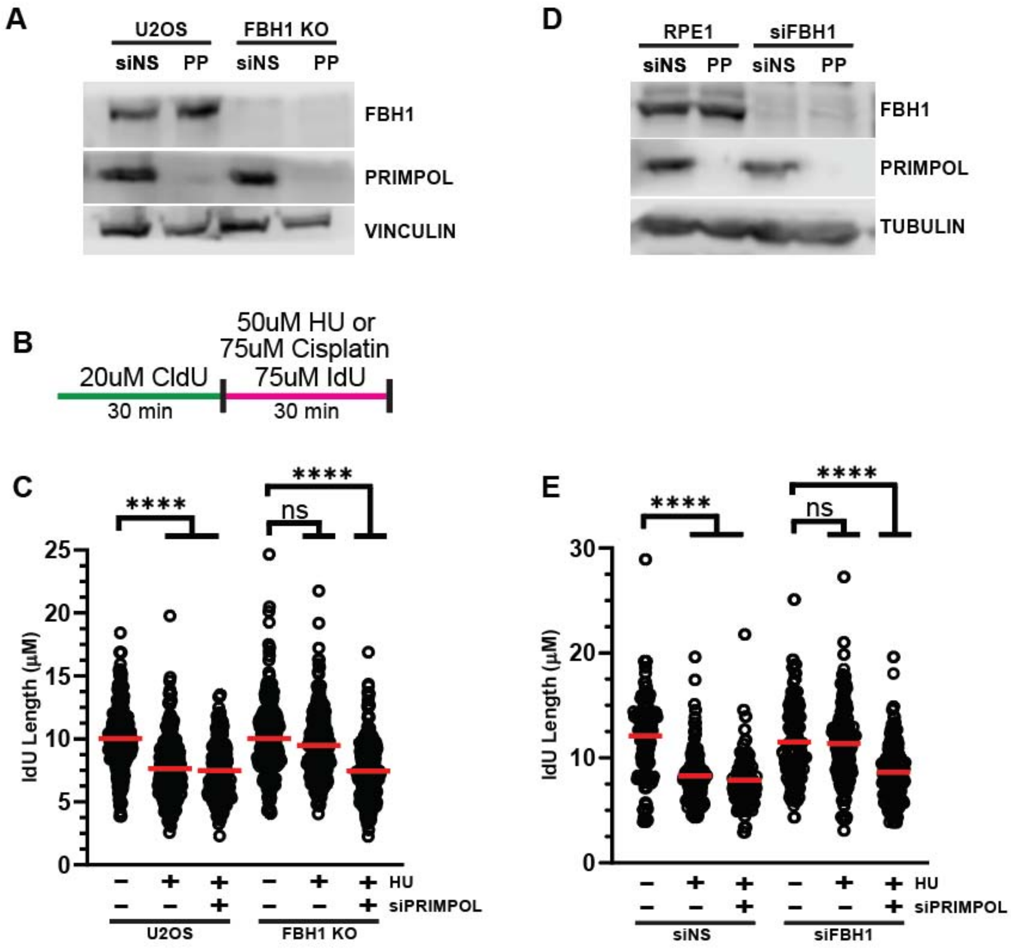

**Figure 1. FBH1 inhibits PRIMPOL-mediated DNA synthesis in response to HU**. **(A)** Western blot depicting FBH1 and PRIMPOL protein levels in U2OS cells. Vinculin is the loading control. **(B)** Schematic of the replication fiber experiment. **(C)** Dot plot depicting IdU tract lengths in U2OS cells after the inducted treatments. At least 200 replication tracts per condition. Red line indicates sample mean. **(D)** Western blot depicting FBH1 and PRIMPOL protein levels in RPE-1 cells. Vinculin is the loading control. **(E)** Dot plot depicting IdU tract lengths in RPE-1 cells after the inducted treatments. At least 200 replication tracts per condition. Red line indicates sample mean. n=3, *****p*<0.0001 ANOVA; Tukey HSD

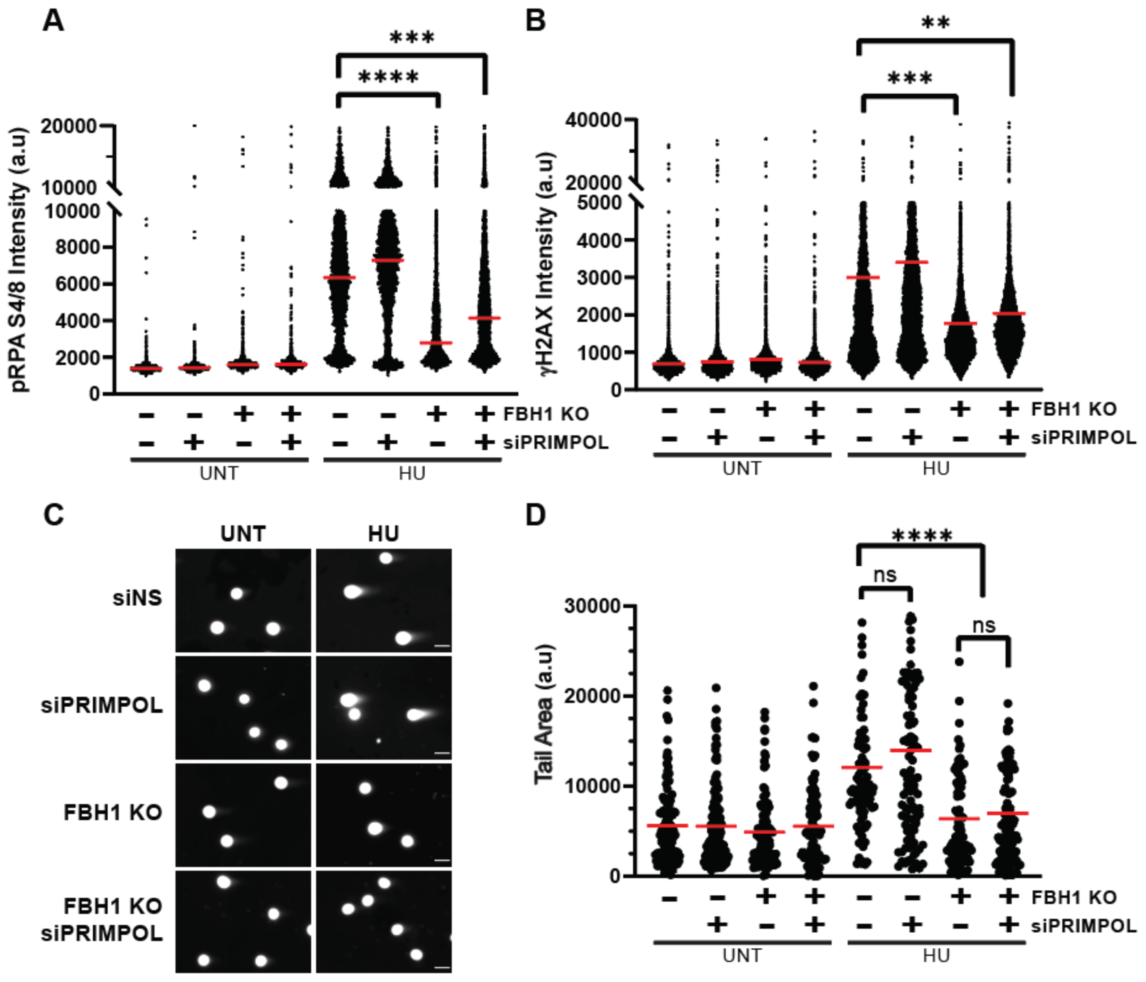

**Figure 2. FBH1 KO prevents DSB formation in a PRIMPOL-independent manner. (A)** Dot plot of at least 3000 nuclei depicting the average pRPA S4/8 intensity per cell. Red line indicates sample mean, **(B)** Dot plot of at least 3000 nuclei depicting the average γH2AX intensity per cell. Red line indicates sample mean **(C)** Representative images of neutral comet assay with DNA stained with SYBR gold (white). Scale bar = 25µm. **(D)** Dot plot of tail area output from neutral comet assay. Red line indicates sample mean. n=3 **$p<0.01$ ***$p<0.001$ ****$p<0.0001$ ANOVA; Tukey HSD

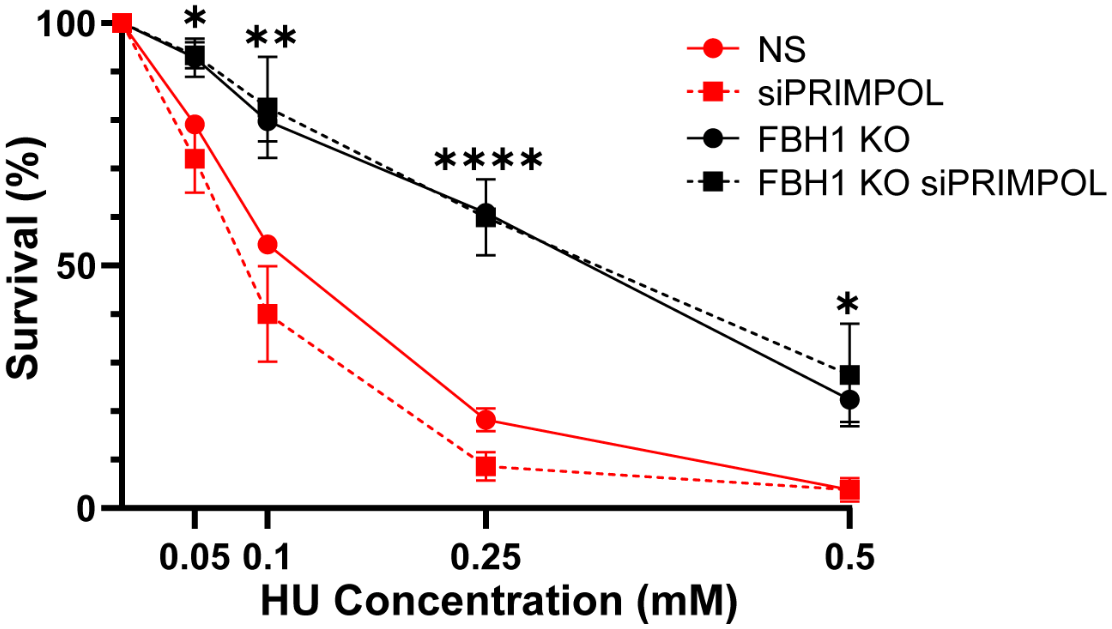

**Figure 3. Unrestrained PRIMPOL does not promote increased HU resistance in FBH1 KO cells**. Survival curve after 48-hour HU treatment and 7-day outgrowth, n=3, *$p<0.05$ **$p<0.01$ ****$p<0.0001$ ANOVA; Tukey HSD
